# A Thirty-one-year Old Pregnant Woman With Infiltrative Cardiac Masses

**DOI:** 10.5812/cardiovascmed.6926

**Published:** 2013-02-24

**Authors:** Zahra Alizadeh-Sani, Kambiz Mozaffari, Zahra Khajali

**Affiliations:** 1 Rajaie Cardiovascular Medical and Research Center, Tehran University of Medical Science, Tehran, IR Iran

**Keywords:** Malignant Tumor, Liposarcoma, Myxoid, Magnetic Resonance Imaging

## Abstract

Primary cardiac tumors are rare in all ages. Their reported prevalence ranges from 0.001 to 0.03 percent in autopsy series. 25 percent of primary cardiac tumors are considered to be malignant, the majority of which are sarcomas. On account of the late presentation of symptoms in malignant heart masses, finding locally infiltrative tumors or systemically widespread cases at initial presentation is common. We present a case of malignant heart tumor in a thirty-one-year old woman who was first examined here after the termination of pregnancy.

## 1. Introduction

Primary cardiac tumors are rare in all ages. Their reported prevalence ranges from 0.001 to 0.03 percent in autopsy series (1). Secondary involvement by extracardiac neoplasms is 20 to 40 times more common (1-3). Despite their rarity, multiple types of primary cardiac tumors have been histologically recognized. The great majority of the primary tumors of the heart are mesenchymal, displaying the full spectrum of differentiation.25 percent of primary cardiac tumors are considered to be malignant, the majority of which are sarcomas. 

On account of the late presentation of symptoms in malignant heart masses, finding locally infiltrative tumors or systemically widespread cases at initial presentation is common. The diagnosis of primary tumors of the heart is often a challenging process. With the advent of noninvasive and relatively sensitive imaging techniques including cardiac echocardiography, computed tomography (CT), and cardiac magnetic resonance (CMR) the identification of cardiac lesions have been greatly facilitated. We present a case of malignant heart tumor in a thirty-one-year old woman who was first examined here after the termination of pregnancy.

## 2. Clinial History

**Figure 1.**
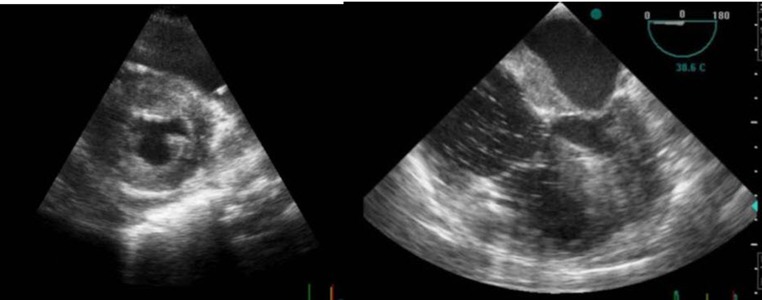
Transthoracic and transesophageal echocardiography showed interatrial and left ventricular thickening

**Figure 2.**
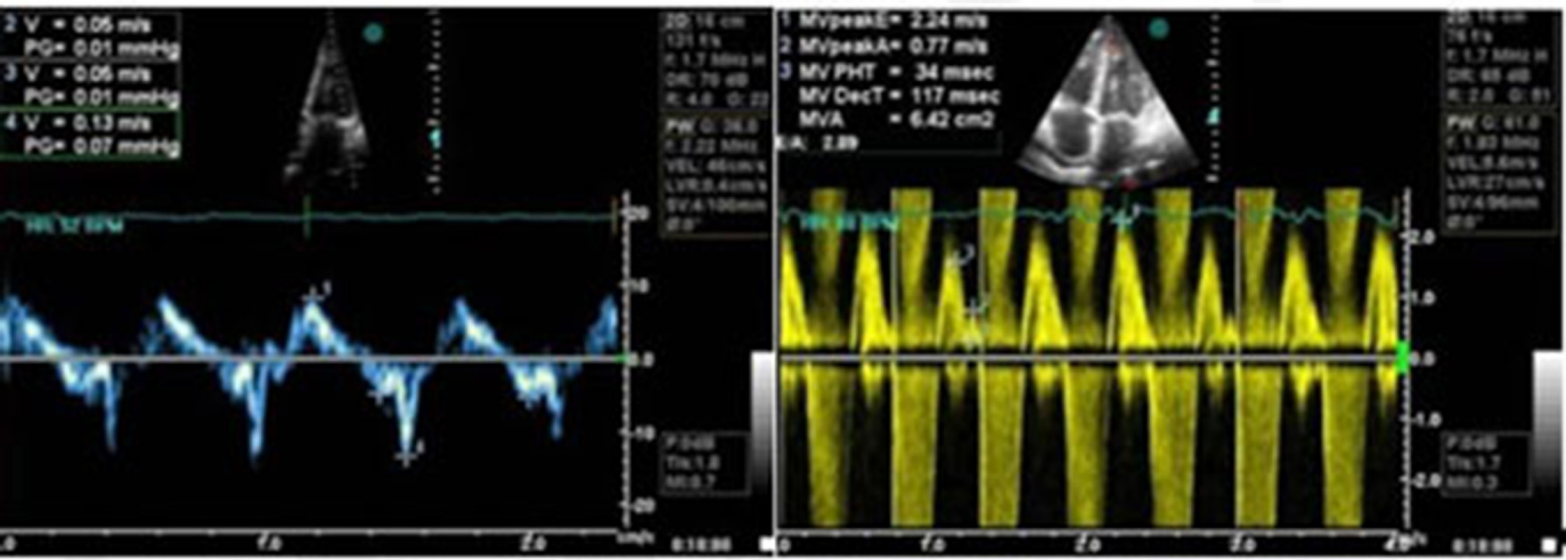
Pulse wave and tissue doppler study in echo showed diastolic dysfunction grade III

A thirty-one-year old woman with progressive dyspnea and lower limbs edema was admitted to another hospital, during the 28th week of gestation. In view of her worsening symptoms, and the presence of a cardiac mass which was discovered by echocardiographic studies, she was recommended to undergo abortion. A stillborn female baby was the result of her Cesarean section. After the termination of her pregnancy, she was referred to our center to further evaluate her cardiac mass. Her physical examination revealed ascites, edema of lower limbs, and tachycardia. The chest X-ray showed mild cardiomegaly with bilateral pleural effusions.Transthoracic and transesophageal echocardiographic studies showed diffuse left ventricular wall thickening with a severe diastolic dysfunction. Biatrial enlargement, thickening of the heart valves with severe mitral and tricuspid valves regurgitation and a homogenous mass measuring 3.2 x 2 cm, which arose from the right upper pulmonary vein, extending to the atrial septum and the anterior mitral valve leaflet were among the other findings (Figures 1 and 2). 

**Figure 3.**
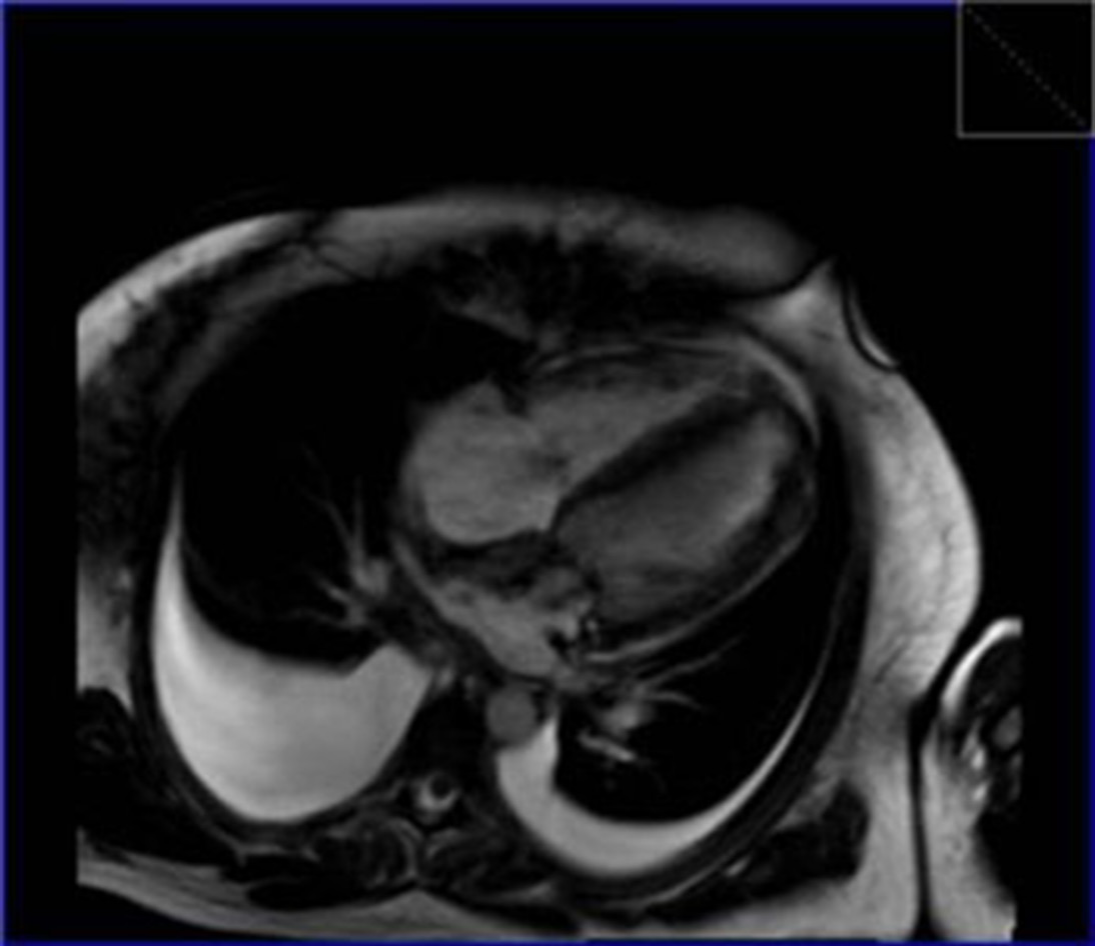
Steady state free precession showed infiltrative nodular mass in both ventricles and interatrial septum

Cardiac MRI was performed to evaluate the extent of the cardiac mass and the possibility of extracardiac tumoral involvement. Steady state free precession (SSFP) images demonstrated diffuse multiple nonhomogenous and infiltrative nodular mass lesions in both ventricles, the interatrial septum with extension to the mitral valve apparatus together with a large cauliflower-like mass which protruded into the right ventricular outflow tract cavity (Figure 3). 

**Figure 4.**
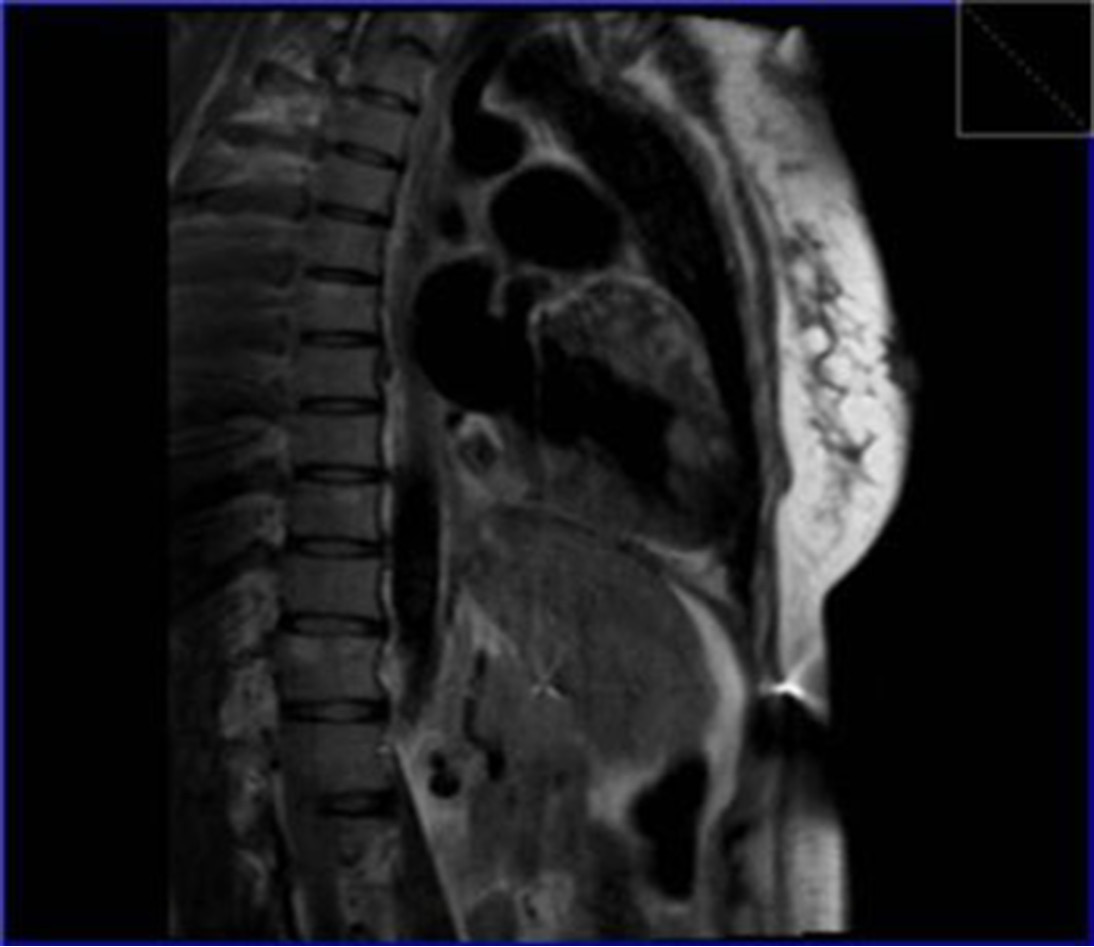
Short-tau inversion recovery and T1 weighted images showed disseminated infiltrative myocardial masses with heterogeneous signal intensity

**Figure 5.**
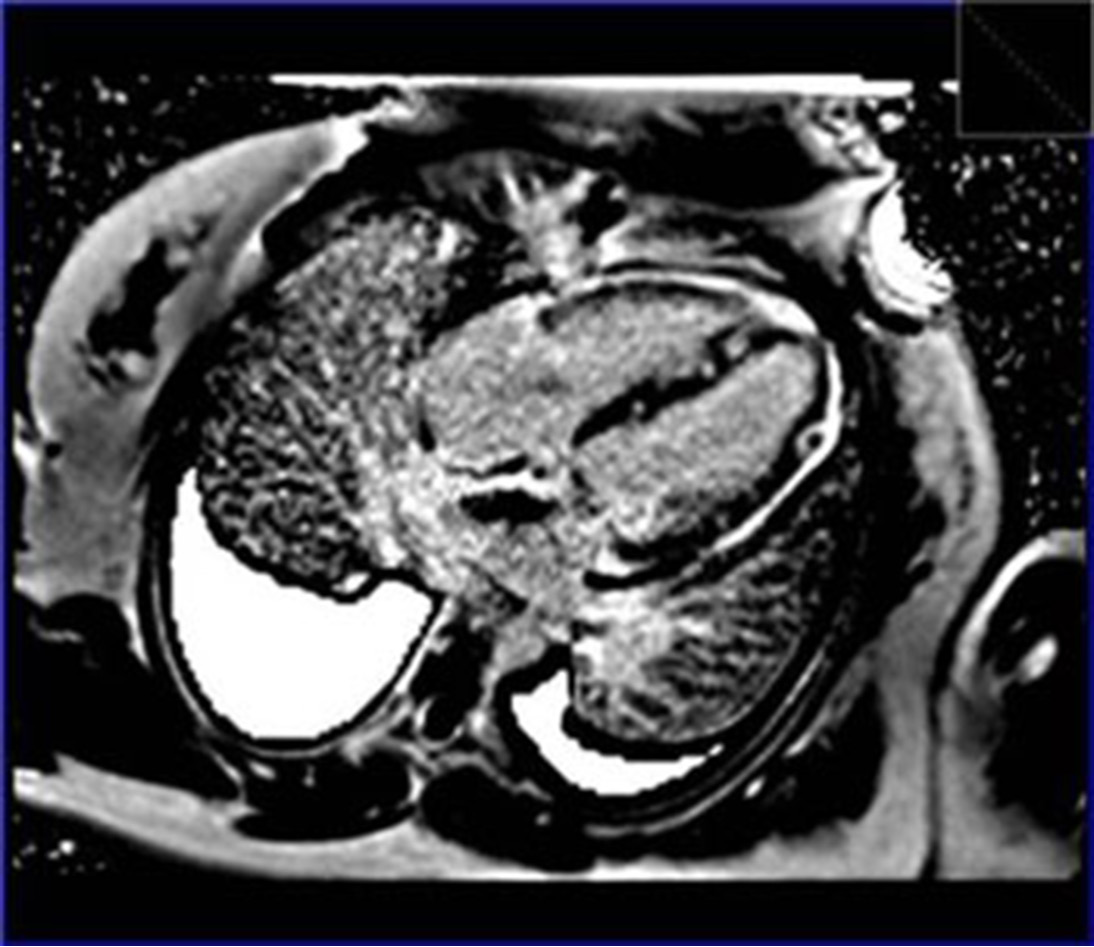
Late gadolinium enhancement showed multiple patchy intramyocardial irregular enhancement

**Figure 6.**
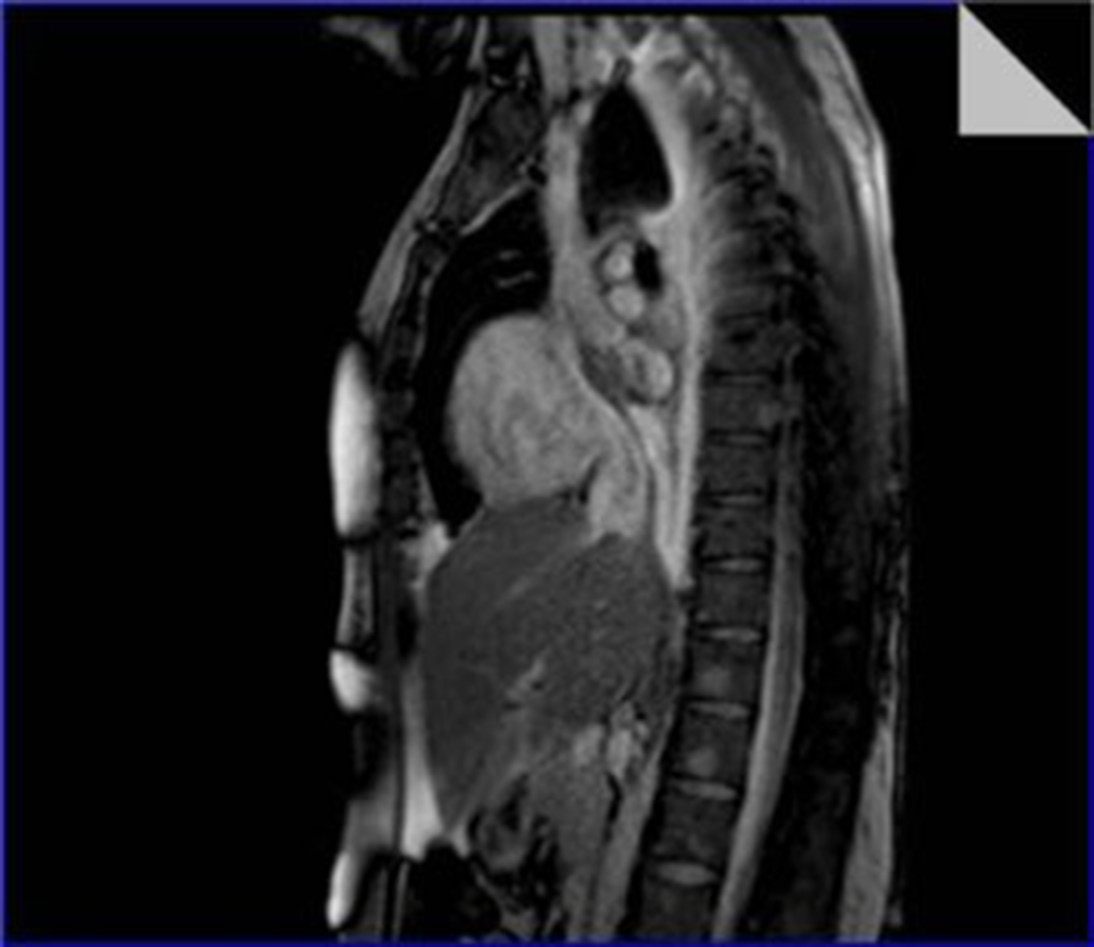
Steady state free precession image showed multiple infiltrative nodular lesions in the thoracolumbar vertebral column

This disseminated mass had nearly isosignal intensity in the T1 weighted images with an increased signal intensity in the short-tau inversion recovery (STIR) sequence (Figure 4) and irregular contrast enhancement in the early and late gadulinum enhancement images (Figure 5) with no evidence of fat component. Multiple nodular lesions in the thoracic vertebral column had also been reported (Figure 6).Bone marrow aspiration cytology from the lytic lesions was done but unfortunately no conclusive data was obtained. The patient underwent a fluoroscopic endomyocardial biopsy which was guided by TEE. The specimen was sent to the histopathology laboratory. 

**Figure 7.**
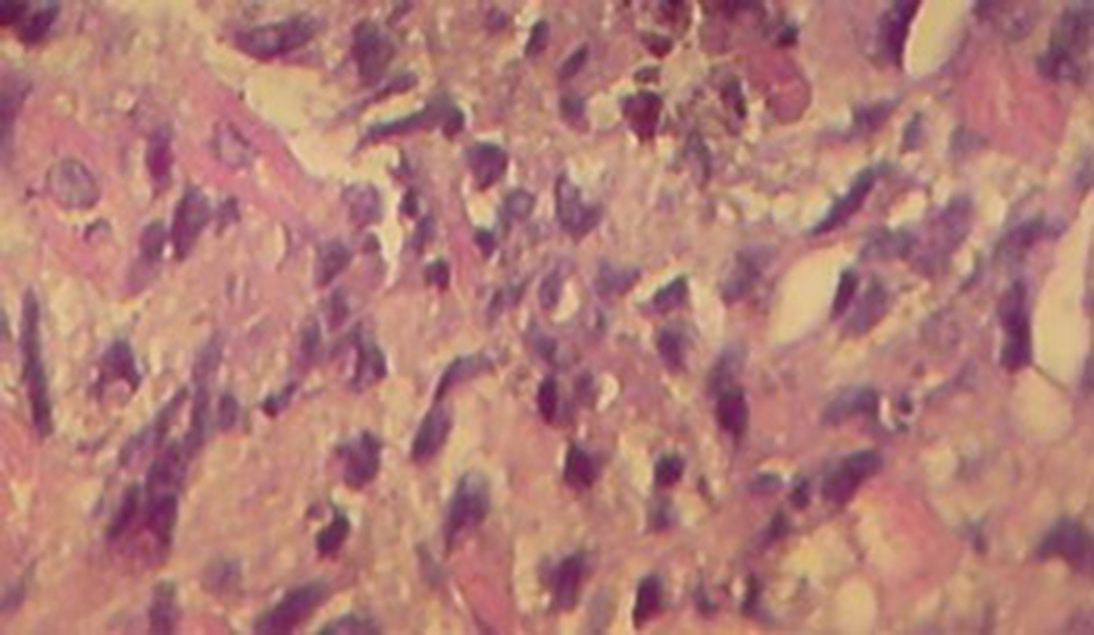
Pathologic specimen: Infiltration of myocardium by a malignant myxoid tumor with moderately pleomorphic cells and round to oval nuclei (H&E X 400)

Grossly, it consisted of tiny creamy fragments of myocardial tissue and some clot. Microscopically, most of the myocardial samples were unremarkable, except for one of the tissue fragments in which myocytes were infiltrated and separated by a myxoid tissue with cells that were moderately pleomorphic and had round to oval nuclei (Figure 7). No bizarre mitotic activity or tissue necrosis could be seen. Given the above circumstances, a diagnosis of myxoid spindle cell sarcoma was established and the clinicians were advised to retake sufficient samples for Immunohistochemistry (IHC) studies. As the patient was a poor surgical candidate, no further diagnostic procedures were deemed advisable. Chemotherapy was supposed to be instituted elsewhere before which, unfortunately the patient died as a result of refractory cardiac arrests in another hospital in her hometown. 

## 3. Discussion

Primary cardiac tumors are rare. Primary cardiac sarcoma (PSC) is a rare and aggressive malignancy that is usually diagnosed late in its course due to its nonspecific symptoms (4). Most patients with primary heart sarcomas present with intractable congestive heart failure, arrhythmias, or signs of superior vena cava obstruction. In rare cases, a metastatic lesion is the first manifestation of the disease (5). Transthoracic echocardiography is the first imaging tool for the diagnostic evaluation of patients with suspected cardiac masses. CMR complements echocardiography in our patient evaluation because of its ability in tissue characterization and wider field of view (6).CMR was used to localize the extent of tumor within the right ventricle and in the cardiac chambers. Regarding CMR results which confirmed the local invasion of the myocardial wall and vertebral metastasis, the absence of fat within the mass, gadolinium perfusion and late gadolinium enhancement we suggested that the nature of the tumor as being malignant. With identification of myocardial infiltration and tumor dissemination by CMR can also help tumor staging and therapeutic management. 

Histopathological studies were indicative of a myxoid sarcoma, however, in view of the fact that tissue samples were scant in amount, confirmation of its histogenesis by immunohistochemical methods were further rendered impossible (3).
